# Targeting microbial quorum sensing: the next frontier to hinder bacterial driven gastrointestinal infections

**DOI:** 10.1080/19490976.2023.2252780

**Published:** 2023-09-07

**Authors:** Ying Su, Tao Ding

**Affiliations:** a Department of Immunology and Microbiology, Zhongshan School of Medicine, Sun Yat-Sen University, Guangzhou, China; b Ministry of Education, Key Laboratory of Tropical Diseases Control (Sun Yat-Sen University), Guangzhou, China

**Keywords:** Gastrointestinal microbiota, gastrointestinal infections, quorum sensing, interspecies and interkingdom cross-talk, quorum sensing interference, live biotherapeutics, microbiota manipulation, anti infection

## Abstract

Bacteria synchronize social behaviors via a cell-cell communication and interaction mechanism termed as quorum sensing (QS). QS has been extensively studied in monocultures and proved to be intensively involved in bacterial virulence and infection. Despite the role QS plays in pathogens during laboratory engineered infections has been proved, the potential functions of QS related to pathogenesis in context of microbial consortia remain poorly understood. In this review, we summarize the basic molecular mechanisms of QS, primarily focusing on pathogenic microbes driving gastrointestinal (GI) infections. We further discuss how GI pathogens disequilibrate the homeostasis of the indigenous microbial consortia, rebuild a realm dominated by pathogens, and interact with host under worsening infectious conditions via pathogen-biased QS signaling. Additionally, we present recent applications and main challenges of manipulating QS network in microbial consortia with the goal of better understanding GI bacterial sociality and facilitating novel therapies targeting bacterial infections.

## Introduction

The human body is a large reservoir of microorganisms that colonize various parts of the body, including the skin, mouth, reproductive organs, and gastrointestinal (GI) tract. These microorganisms, primarily bacteria, have a close relationship with the human host and play a crucial role in maintaining a healthy state.^1^ Particularly in the GI tract, these commensal microbes perform important functions such as providing nutrients to the host, developing the immune system, and preventing the colonization of pathogenic microbes.^2^ The proper structure of the GI microbiota is essential for healthy gut functions, which rely on effective cell-cell communication and interaction. Quorum sensing (QS) is a universal communication system used by bacteria to coordinate social behaviors that shape the structure and function of polymicrobial communities. QS triggers changes in global gene expression by responding to different autoinducers (AIs) secreted by bacteria or even the host.^3–6^ The accumulation of AIs in the human microbiota is closely associated with the bacterial composition. When a bacterial infection occurs in the human body, the invading pathogens disrupt the intrinsic balance of the signaling network and deploy their own AI signals to recruit other synergistic accomplices, forming denser polymicrobial biofilms and increasing toxin production, thereby worsening the infectious situation.^7,8^

Quorum sensing systems have been identified in various bacteria isolated from the human body, including pathogenic bacteria such as *Fusobacterial nucleaum*,^9^ *Helicobacter pylori*,^10^ *Escherichia coli*,^11^ *Salmonella enterica*,^12^ and probiotics like *lactobacillus*. ^13^ QS signaling systems have been extensively studied in these individual bacteria and have been shown to be involved in various processes related to bacterial colonization, virulence expression, and biofilm formation. However, it is still challenging to understand how these systems work together to establish an effective bacterial communication network and maintain bacterial community homeostasis in multispecies consortiums like the human gut microflora. Infection models have provided valuable insights into the QS-regulated social behaviors of bacteria in the context of multispecies communities. In this review, we will discuss the basic molecular mechanisms of QS in infectious pathogens and highlight the role of QS in exacerbating infectious conditions. Our aim is to demonstrate how these studies contribute to our current understanding of QS in bacteria associated with the human host, both in terms of fundamental knowledge and practical applications.

## Classical autoinducers identified in GI microbiota

Quorum sensing (QS) is a regulatory mechanism that controls sporulation, biofilm production, virulence secretion, and interactive relationships of bacteria, including interspecies competition, cooperation, and kinship inference.^14–16^ QS relies on the production, release, and detection of extracellular signaling molecules called autoinducers (AIs). Gram-negative (Gram^−^) bacteria primarily use species-specific acyl-homoserine lactone (AHL) as their AI, while Gram-positive (Gram^+^) bacteria mainly use autoinducing peptides (AIPs). Another universal language used by both Gram^+^ and Gram^−^ bacteria is autoinducer-2 (AI-2), also known as furanosyl borate diester or tetrahydroxy furan. While numerous studies have revealed the regulatory role of bacterial AIs in controlling physiological functions in certain bacteria, we are particularly interested in exploring the role of bacterial AIs in inter-species and inter-kingdom communication and interaction, especially in the human GI tract.

### AHL-based signaling facilitates bacterial adaptation in the GI tract

AHLs are the most common AIs utilized by Gram^−^ bacteria.^17^ These compounds consist of a core *N*-acylated homoserine lactone ring and a fatty acid chain of 4–18 carbon atoms in length. Moreover, AHLs can have various modifications on their acyl chains, adding further diversity to the signaling molecules.^18^ AHLs are synthesized by LuxI type synthases via deriving the lactone moiety, an intermediate of fatty acid biosynthesis, from S-adenosylmethionine (SAM), and sensed by LuxR type receptor, a cytoplasmic transcription factor with two functional domains for AHL and DNA binding.^19^ Usually, the LuxI and LuxR are coexisted in human pathogens, such as *Pseudomonas aeruginosa*,^20^ *Yersinia enterocolitica* ^21^ and *Burkholderia cepacia*,^22^ and they are crucial to produce virulence factors, colonization and infection, biofilm formation and antimicrobial resistance.^23–25^

**Figure 1.**
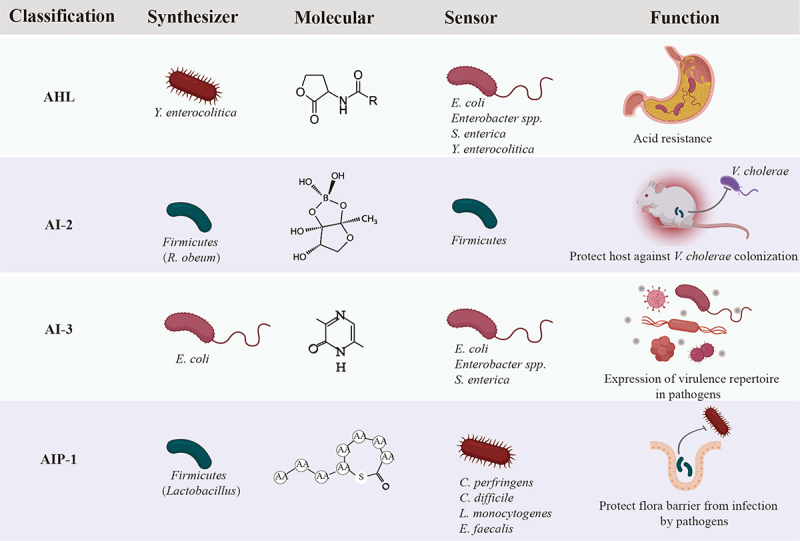
Classical autoinducers (AIs) identified in GI microbiota and examples of the regulatory role of these AIs in controlling physiological functions in GI bacteria.

AHL type molecules have been detected in sputum and saliva of infected patients,^26,27^ infected wounds^28^ and human feces of gastrointestinal disease patients as well as healthy subjects,^29,30^ which suggests a potential regulatory role of AHLs playing in human GI microbiota. Although the evidence of the AHL production by commensal bacteria are still lacking, however, there exist quantities of bacteria in the human gut possessing LuxR solos/orhpans, such as *E. coli*, *S. enterica*, *Enterobacter* spp., and *Klebsiella pneumoniae*.^31^ These bacteria encode a LuxR homolog named SdiA, but do not encode a partner LuxI homolog or synthesize their own AHLs, which enables them to sense and respond to AHLs produced by other members in the microbiota. A recent study investigated the presence and expression of AHL synthases and receptor genes in human gut during IBD by *in silico* approach.^32^ Although no known AHL synthase was identified, orphan LuxR homologs in human gut bacteria were observed and reported differential expressions in different Bacteroides in IBD against non-IBD group of people.^32^ Perceiving exogenic AHLs might be an advanced survival strategy for bacteria, which facilitates them to sense and adjust to the changeable living environment. For example, an enterohemorrhagic *E. coli* (EHEC) O157:H7 would activate gene expression related with acid resistance when sensing surrounding AHLs.^33,34^ This may be the reason explaining how EHEC survive when passed through bovine acidic stomachs (Figure 1).

### AI-2 based signaling maintains the homeostasis of microbial barrier to against pathogen invasion

AI-2 molecules are widely accepted as a universal bacterial language allowing for interspecies communications and interactions. They are encoded by LuxS, which is ubiquitous and conserved in both Gram^+^ and Gram^−^ bacteria.^35^ Moreover, AI-2 molecules have also been detected in stools from healthy human, suggesting that AI-2 is normally present in human GI tract.^36^

GI bacteria are expected to utilize AI-2 strengthening the beneficial interactions, thus structure homeostatic bacterial consortia and protect host against colonization of pathogens. It has been demonstrated that antibiotic treatment to mice would eliminate Firmicute in their intestine and increase the infection risk by *Salmonella enterica* serovar Typhimurium.^37,38^ Thereafter, the author introduces an engineered *E. coli* to the mice aiming in restoring the concentration of AI-2 in the mouse gut. The test result shows that the introduced *E. coli* increases intestinal AI-2 level, thereafter favors the expansion of Firmicutes phylum in the mouse gut.^38^ This suggests the regulating role of AI-2 in maintaining homeostasis of GI flora. Besides, AI-2 can also strengthen intestinal barrier against the invasion by pathogens. As demonstrated by Hsiao, AI-2 produced by *Ruminococcus obeum*, a commensal GI bacterium, can protect mice against *V. cholerae* colonization (Figure 1).^39^ It is interesting to note that the expression of *luxS* gene in *R. obeum* was upregulated in response to *V. cholerae*, which reflects an immediate reaction on signaling communication by commensals when they get threated by unexpected invaders.

### Competition between symbiotic and pathogenic bacteria based on AIP

AIP is a series of small, secreted peptides, mainly deployed by Gram^+^ for QS signaling. These peptides are encoded as a precursor from the QS operon, then processed and secreted by a specialized system named ATP-binding cassette transporter. Secreted AIPs accumulate with an increase of the bacteria density, subsequently be detected by the two-component sensor histidine kinases, such as *Agr* system in *Streptococcus* and Firmicutes.^17,40^ Interplay between AIP and sensor kinase would onset the phosphorylation of the cytoplasmic response regulator that controls the transcription of QS-regulated genes.

The interplay between AIP and its receptors is strongly species specific, that is, the non-cognate AIP can inhibit QS signaling in other strains.^41,42^ One recent study presents that AIPs produced by *Agr* system of commensal *Staphylococcus simulans* block the QS system in a methicillin-resistant strain *Staphylococcus aureus*, therefore protects host skin from damage.^43^ The competing strategy via AIP is likely prevalent in GI microbiota, considering that the *Agr* system is evolutionarily ubiquitous across Firmicutes, including probiotics like *Lactobacillus plantarum* as well as human pathogens like *Clostridium perfringens*, *Clostridium botulinum*, *Clostridium difficile*, *Listeria monocytogenes* and *Enterococcus faecalis* (Figure 1).^44^ We envision that AIP signaling by commensal bacteria is another key factor influencing the structure and function of GI microbiota. Antibiotic treatment dramatically decreased the abundance of Firmicutes in mammalian gut,^37,38^ which means the intrinsic QS architectures and protective flora barrier would be destroyed. The interfered QS signaling can be recognized by pathogens and accelerate the colonization by them. That is one of the key reasons contributing to the severe risk of infection by *C. difficile* and *S. typhimurium* after antibiotic treatment.

### AI-3 based signaling in the GI tract

AI-3 is another constantly discovered QS signal in GI tract. This chemical is synthesized by bacterial members in human GI flora, such as enterohemorrhagic *E. coli*. ^36,45^ Although the pathway of AI-3 synthesis is still less defined, the threonine dehydrogenase (Tdh) and the aminoacyl-tRNA synthetases-related spontaneous cyclization are identified to be essential for AI-3 production.^45^ AI-3 signal can be sensed by *E. coli*, *S. typhimurium*, and *Enterococcus* via a histidine kinase sensor QseC to regulate the expression of their virulence repertoire (Figure 1).^36,46^ Including AI-3, QseC can sense host hormones like epinephrine (Epi) and norepinephrine (NE), which indicates a manipulating ability of host on GI microbiota via mammalian signal Epi/NE.^47^ However, AI-3 and its analogs do not affect adrenergic signaling in human cells *in vitro*.^45^

## The effect of microbiota plays on host driven by QS

**Figure 2.**
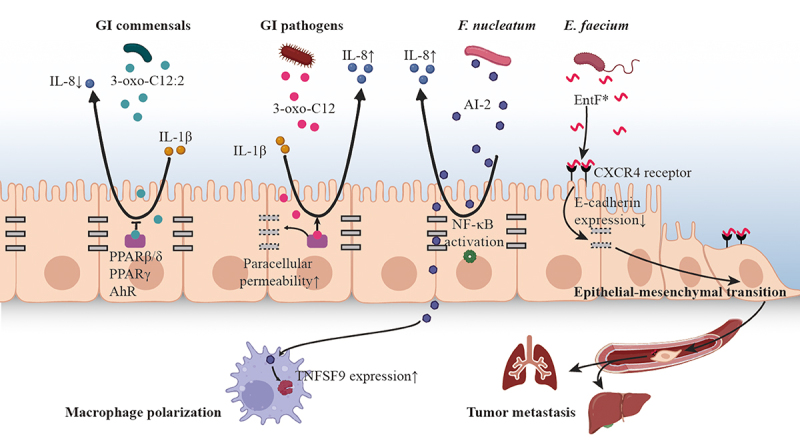
The effect of microbiota plays on host driven by QS.

Evidence has shown that AI molecules such as AHL, AI-2 and AIP, which are frequently found in GI microbiota, can interact with intestinal mucosal cells and affect the inflammation and carcinogenesis process of host (Figure 2) (Table 1). On the other hand, perceiving AIs from the parasitic microbiota enables host to continuously monitor bacterial communications and bacterial infection dynamics. Clarifying the interkingdom interactions from the perspective of QS are essential for maintaining the homeostasis of the microbiota as well as the health of the host.

3-oxo-C12:2 and 3-oxo-C12 (AHL type) show opposite effect on IL-8 secretion in IL-1β-stimulated. PPARβ/δ, PPARγ and AhR are suspected to be putative mammalian AHL receptors, participating the expression of proinflammatory genes. AI-2 activates the NF-κB signaling pathway to upregulate the expression of IL-8. AI-2 promotes macrophage polarization via stimulating the expression of TNFSF9. EntF* (AIP) promotes epithelial-mescenchymal transition via inhibits the expression of E-cadherin. EntF* facilitates colorectal cancer metastasis with metastatic lesions found in both liver and lung tissues, using an orthotopic mice model.

**Table 1.** QS mediated inter-species and inter-kingdom communications in GI tract (mammalian host).

Type	QS signal	Singal source		Receptor	Function	Model	References
Interspecies interaction	AHL	Unidentified commensal bacteria in bovine rumen	Prokaryote	Enterohemorrhagic *Escherichia coli* via SdiA	Activate genes involved in acid resistance in *E. coli* (EHEC) O157:H7	/	^33,48^
	AI-2	Engineered *E. coli*	Prokaryote	Firmicutes phylum	Recover the abundance of Firmicutes phylum eliminated by streptomycin in the gut of mice	*In vivo* mice model	^38^
	AI-2	*Ruminococcus obeum*	Prokaryote	Vibrio cholerae	Inhibits virulence gene expression by *V. cholerae* and restricts *V. cholerae* colonization in mice	*In vivo* mice model	^39^
	AIP	*Staphylococcus simulans*	Prokaryote	Methicillin-resistant S. aureus	Reduce dermonecrotic and epicutaneous skin injury in murine models caused by *S. aureus*	*In vivo* mice model	^43^
	AI-3	GI microbiota	Prokaryote	E. coli, Salmonella enterica serovar Typhimurium, and Enterococcus	Regulate expression of bacterial virulence genes	*In vitro* experiment and *in vivo* rabbit model	^36,46^
Interkingdom interaction	3OC12-HSL	Chemically synthesized molecule	Prokaryote	Mammalian cells	Stimulate a significant induction of mRNAs for the cytokines IL-1α (IL-1α) and IL-6	*In vivo* mice model	^49^
	3OC12-HSL	Chemically synthesized molecule	Prokaryote	Mammalian cells	Suppress immune activity by inhibiting cytokine production and lymphocyte proliferation, disrupting NF-κB functions, and inducing immune cell death	*In vivo* mice model	^50–53^
	3OC12:2-HSL	Gut microbiota	Prokaryote	Caco-2 cells	Suppress inflammatory effect on Caco-2 cells	*In vitro* experiment	^30^
	AI-2	In vitro-synthesized signal molecule	Prokaryote	HCT-8 colon cancer cells	Induce the production of inflammatory cytokines like intereukin-8	*In vitro* experiment	^54^
	AI-2	*F. nucleatum*	Prokaryote	Macrophages	Enhance the mobility and M1 polarization of macrophages by activating the TNFSF9/IL-1β pathway	*In vitro* experiment	^55^
	AI-2	*H. pylori*	Prokaryote	Gastric epithelial cells	Inhibit the bacterial adhesion, expression, and translocation of CagA, and attenuates the inflammatory response of AGS cells induced by H. pylor	*In vitro* experiment	^56^
	AIP	*Bacillus* sp., *Enterococcus faecium* and *Escherichia coli*	Prokaryote	HCT-8/E11 colon cancer cell	Initiate HCT-8/E11 colon cancer cell invasion	*In vitro* experiment	^57,58^
	Phr0662	*Bacillus* sp.	Prokaryote	Chick chorioallantoic membrane with tumor cells	Promote tumor angiogenesis	*In vitro* experiment	^59^
	EntF*	*E. faecium*	Prokaryote	Mouse	Promote colorectal cancer metastasis with Metastatic lesions found in both liver and lung tissues	*In vivo* mice model	^60^
	AI-2 mimics	Mammalian epithelial cells	Eukaryote	Bacteria possessing AI-2 receptor LuxP/LsrB, such as enteric pathogen Salmonella typhimurium	Activates QS-controlled gene expression in *S. typhimurium*	*In vitro* experiment	^61^
	Epinephrine and norepinephrine (AI-3 mimics)	The adrenal medulla and sympathetic adrenergic neurons in gut	Eukaryote	Salmonella enterica and E. coli via QesC	Affect the signal reception of AI-3 and activate the virulence of *E. coli* O157:H7 and *Salmonella;* a *qseC* mutant is attenuated for virulence in a rabbit animal model	*In vitro* experiment and *in vivo* rabbit model	^45,46^
	Dynorphin	Mice intestinal mucosa	Eukaryote	P. aeruginosa	Activate QS signaling in *P. aeruginosa* and enhances its virulence *in vivo*	*In vivo* mice model	^62^
	PON enzyme family (AHL lactonase)	Eukaryotic cells	Eukaryote	P. aeruginosa	Subverts P. aeruginosa QS and biofilm formation *in vitro*; protectes *Drosophila melanogaster* from *P. aeruginosa* lethality *in vivo*	*In vitro* and *in vivo* experiments	^63,64^

### AHL-elicited immunomodulatory activity

AHLs are fatty acid-based signaling molecules that are chemically similar to lipidic and steroid hormones in eukaryotes.^65^ As one of the signaling molecules produced by the infamous *P*. *aeruginosa*, 3-oxo-dodecanoyl-l-homoserine lactone (3OC12-HSL) has been frequently detected from sputum, saliva and feces collected from human host,^26,27,29^ and its role in inter-kingdom signaling has been fully studied. Injection of 3OC12-HSL into mouse skin increases the expression of cytokines IL-1α and IL-6, which in turn leads to subsequent inflammation of the host.^49^ On the other hand, this molecule can suppress immune activity by inhibiting cytokine production and lymphocyte proliferation,^50,51^ disrupting NF-κB functions,^52^ inducing immune cell death^53^ and decreasing antibody responses in mammalian cells.^66^ A newly discovered AHL in human gut, 3-oxo-C12:2 is significantly more frequent in fecal samples from healthy subjects comparing with that in IBD patients. This molecule exerts an anti-inflammatory effect on Caco-2 cells by decreasing IL-1β induced IL-8 secretion. In contrast, 3OC12-HSL increases paracellular permeability but 3-oxo-C12:2 does not (Figure 2), which indicates the protective role of 3-oxo-C12:2 plays on both gut microbiota and intestinal epithelial cells.

Receptors for AHL and related signaling pathways in mammalian host are still poorly understood. Nevertheless, the peroxisome proliferators-activated receptor PPARβ/δ and PPARγ are suspected to be putative mammalian 3OC12-HSL receptors, participating the expression of proinflammatory genes.^67^ Another host receptor, aryl hydrocarbon receptor (AhR), can detect the type and quantity of quorum-sensing molecules of *P. aeruginosa* including AHL, quinolones, and phenazines (Figure 2). Through the recognition of different signal molecules by AhR, the host judges the degree of bacterial infection, thereafter adjust the immunologic response.^68^ This mechanism may explain why an opportunistic pathogen can be tolerated by the host at low density but become harmful once a threshold of tolerability has been exceeded. Host receptors having similar function to AhR may widely existed, therefore the immunologic defense would be specifically focused on harmful traits instead of harmless traits, allowing the host to mobilize the most appropriate defense mechanism according to the severity of threat.

### Proinflammatory effects caused by AI-2

AI-2 is also involved in inflammatory process of host cells as a response to a challenge with bacterial pathogens. AI-2 is able to affect the proinflammatory responses of host to GI pathogens, like *F. nucleatum*,^69^ *P. gingivalis*,^70^ and *H. pylori*.^56^ The AI-2 concentration in both colorectal tissue and stool of CRC patients was significantly higher compared with that in colorectal adenoma (AD) and normal colon mucosa (NC),^71^ witch suggests an intense interaction between the secreted AI-2 and the immunity of colorectal cancer (CRC). An *in vitro* study reveals that the AI-2 signaling molecule elicits inflammatory by inducing IL-8 expression in HCT-8 colon cancer cells (Figure 2).^54^ Moreover, AI-2 extracted from *F. nucleatum* promotes macrophage polarization via stimulating the expression of TNFSF9 (Figure 2), which was mainly derived from the tumor microenvironment.^71^ This result suggests the AI-2 produced by gut microbiota may play a role in the carcinogenesis of CRC through immune cells of the gut.^71^

Although the above-mentioned research has demonstrated that AI-2 can be produced by the GI microbiota and exists in the intestinal environment, it can also be sensed by intestinal epithelial cells and immune cells, triggering intracellular signal transduction pathways that influence immune responses. However, details regarding which receptors in host cells, particularly intestinal epithelial cells, can recognize AI-2 signal molecules remain uncertain.

### AIP participate in promoting metastasis of cancer cells

The autoinducing peptides (AIPs) synthesized by human microbiota were found to influence mammalian cells (Table 1), such as promoting metastasis of cancer cells^59,60^ and penetrating the blood-brain barrier.^59^ Certain AIP, such as Phr0662 of *Bacillus* sp., EntF-metabolite of *Enterococcus faecium*, and EDF-derived peptides of *E. coli*, could initiate HCT-8/E11 colon cancer cell invasion, with Phr0662 also promoting angiogenesis.^57^ Taking the QS peptide EntF* produced by *E. faecium* as an example, it is found to be naturally present in mice bloodstream. Its role in promoting colorectal cancer metastasis was also observed *in vivo*, with metastatic lesions found in both liver and lung tissues, using an orthotopic mice model. Further *in vitro* tests suggest that EntF* can be sensed by CXCR4 receptors, thereafter inhibits the expression of E-cadherin in the colorectal cells and consequently results in the epithelial-mesenchymal transition (Figure 2). This is likely to be one of the main mechanisms that pathogens promote tumor metastasis.^60^ In addition to CXCR4 receptors, the epidermal growth factor receptor (EGFR) overexpressed in many types of cancer cells are also potential receptors responsible for binding with AIP and activating intracellular signaling cascade that resulting in tumor metastasis.^57^

At present, it is still in the early stage to explore the relationship among QS signaling, intestinal microbiota homeostasis and human health. It is imperative to clarify the characteristics of QS signaling (i.e., type and concentration of AIs) under different health conditions, which will provide us with new concept for disease prevention, diagnosis, and therapy by selective modulation on the gut microbiota.

## The fight back against bacterial QS signaling by host

### Host-mediated AHL degrading and modifying

**Figure 3.**
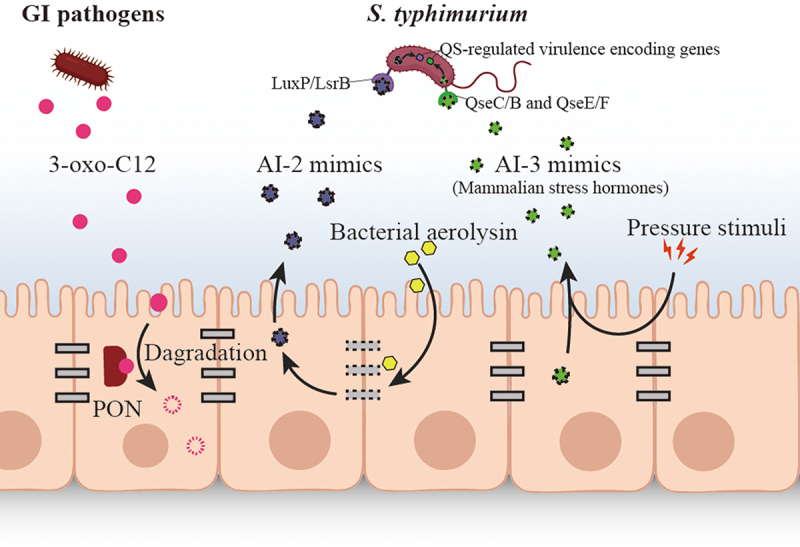
The fight back against bacterial QS signaling by host.

To disrupt bacterial QS, many bacteria have developed the ability to interfere with or manipulate of QS pathways, which termed as quorum quenching (QQ). In multispecies microflora, lactonase, acylase and oxidoreductase are the most frequently investigated enzymes to inactivate AHLs by bacteria. Intriguingly, the eukaryotic cytoplasmic paraoxonases (PONs) can act as lactonases, being able to degrade AHL 3-oxo-C12 (Figure 3).^72,73^ The PON family (PON1–3) was initially proved to subvert the QS signaling of *P. aeruginosa* and its biofilm formation *in vitro*.^63,64^ Later, Stoltz proved the degrading function on AHL by PON1 using a transgenic *Drosophila melanogaster* overexpressing the enzyme, which protect the host from *P. aeruginosa* lethality due to the lactonase activity of PON1 (Table 1).^74^ Another modification mechanism was found in *Hydra vulgaris*, which can modify 3-oxo-C12 into the 3-OH-C12 counterparts, resulting in a phenotypic switch in their bacterial colonizers.^75^ Given *Hydra* is far less advanced than mammals, we suspect the modification of bacterial signals may also exist in the more sophisticated human host. In this way, the host can control or modify the behaviors of its bacterial colonizers for its own benefit.

The eukaryotic cytoplasmic paraoxonases (PONs) can act as lactonases, being able to degrade AHL 3-oxo-C12. Mammalian epithelial cells from colon tissues (Caco-2), lung tissues (A549), and cervical tissues (HeLa) can produce AI-2 mimics in response to bacteria, which can be detected by AI-2 receptors and activate S-controlled gene expression in the enteric pathogen *S. typhimurium*. Hormones E and NE are reported to signal through the same pathway in bacteria with QS signal AI-3, a two-component systems QseC/B and QseE/F. They can activate the virulence of *Salmonella* and *Escherichia coli* O157:H7.

### Synthesis of QS signal mimics by host cells

#### AI-2 mimics

Interkingdom communication between eukaryotic cells and bacteria can rely on the AI-2-based QS. Mammalian epithelial cells from colon tissues (Caco-2), lung tissues (A549), and cervical tissues (HeLa) can produce AI-2 mimics in response to bacteria.^61^ These AI-2 mimics can be detected by traditional AI-2 receptors, LuxP/LsrB, and activate QS-controlled gene expression in the enteric pathogen *S. typhimurium* (Figure 3) (Table 1).^61^ Considering that AI-2 is a universal nonspecific QS signal, synthesizing AI-2 mimics could be a strategy for host to maximally manipulate bacterial behaviors in a mixed population like GI microbiota.

#### AI-3 mimics

Gut hormones such as epinephrine (E) and norepinephrine (NE) that contribute to gut motility, potassium and chloride secretion, epithelial barrier function, and inflammation,^76,77^ can also be perceived by microbial QS receptors and interfere with bacterial behaviors. Hormones E and NE are reported to signal through the same pathway in bacteria with QS signal AI-3, a two-component systems QseC/B and QseE/F.^36,46,78–80^ E and NE are reported to be the host-derived AI-3 mimics, which can affect the signal reception of AI-3 and activate the virulence of *Escherichia coli* O157:H7 and *Salmonella* (Figure 3) (Table 1).^45,46^ Another mammalian stress hormone dynorphin in mice intestinal mucosa was reported to activate QS signaling in *P. aeruginosa*, and therefore enhanced its virulence *in vivo*.^62^ Although there is still limited number of *in vivo* studies about cross-talk between host and bacteria, we expect more *in vivo* evidence to explain the underlying mechanisms.

## QS functions as allay or enemy to host?

### The role of QS in the construction of microbial barrier

Although there are much more studies presenting the role QS plays in pathogenicity, evidence proving QS signaling in commensal flora is arising. The human intestine hosts trillions of microorganisms, which construct a complex microbial community playing multiple anti-infectious, anti-inflammatory, and immune modulating roles decisive for intestinal homeostasis.^81^ In healthy human gut, the commensal flora has attached to the intestinal mucus, occupying all available spaces. They are suspected to recognize and cooperate with each other via QS systems, and then construct a protective barrier against enteropathogenic colonization. Recent study has shown that AI-2 produced by the commensal *R.obeum* inhibits virulence gene expression by *Vibrio cholerae* and restricts *V. cholerae* colonization in mice (Figure 1).^39^ In another study, the Firmicutes phylum previously eliminated by streptomycin was recovered by replenishing AI-2 in the mouse gut.^38^ This demonstrates that AI-2 has great potential assisting commensal bacteria to maintain the homeostasis of gut microbiota, the balance of which is known to influence human health. AI-2 signaling has been identified in the probiotics *E coli* Nissle 1917, *Bifidobacterium* and *Lactobacillus* species, allowing adhesion and enrichment of them in intestines by regulating biofilm formation.^82–84^ However, its role in maintaining homeostasis of commensal flora is still lacking evidence *in vivo*. In addition, other type QS signal derived from commensals (like 3-oxo-C12:2) in strengthening commensal intestinal bacteria cooperation to resist colonization by invaders, and keeping the dynamic balance of the microbiota also requires further exploration.

### How QS accelerate dysbiosis of microbiota during infection

As previous mentioned, the dynamic balance and symbiotic cooperation in GI microbiota is protected by QS. However, when the intrinsic concordant QS network of commensal microbiota gets interfered or impaired due to alterations of host diet, physiology, and immunological processes, the resident pathobionts or external entero-pathogens are highly likely to multiply uncontrollably. In this unbalanced microbiome environment, enteropathogens, such as *E. coli, Clostridia, Listeria, and Pseudomonas species*, utilize their own QS systems to strengthen colonization and invasion of the intestinal mucus layer, thereby promoting bacterial virulence expression, host inflammation, systemic translocation, and ultimately leading to GI or systemic infections.^85–88^

#### Colonization attraction

Some pathogens take advantages of host and microbiota-derived QS signals to selectively colonize in gut. Usually, epithelia cells of intestine directly interact with colonizing bacteria, protecting host from pathogen invading. But, when treated with stress signal, like the bacterial aerolysin, the epithelial tight junctions will be disrupted, and activate production of an AI-2 mimic, which is can activate QS to regulate mechanisms in enteric pathogens, such as *S. typhimurium* (Figure 3).^61^ The release of mammalian AI-2 mimic after epithelial tight-junction damage may call for the cell healing, such as the production of a polysaccharide called PSA, which is an ameliorative bacterially-produced molecule by intestinal commensals.^89^ Besides that, the pathogenic residents that possessing bacterial AI-2 receptors, such as *F. nucleaum*, *C. difficile*, and *E. coli*, are also supposed to sense the AI-2 mimic,^61^ thereby aggregate on the epithelial tight-junction damaging site and lead to an aggravating damage of intestinal barrier. Mammalian stress hormones, epinephrine (Epi), noradrenaline (NE), and dynorphin that induced by stress can affect the response of GI system, including the susceptibility of GI microbiota to pathogenic bacteria.^45,46^ As shown in an *in vivo* experiment, the dopamine β-hydroxylase knockout (*Dbh*^−/−^) disabled in producing Epi or NE, exhibits reduced susceptibility to *S. typhimurium* infection, which proved the attracting function of several host metabolites to enteric pathogens.^90^ Similar attraction effect has been observed in *P. aeruginosa* by dynorphin in colonizing in mice intestinal mucosa.^47^

#### Bacteria coaggregation and polymicrobial biofilm formation

Most of pathogens well characterized so far have QS signaling systems, that is, the reason why pathogens may precisely anchor to the impairing sites of the epithelial barrier based on signals released by cell damage. Especially when commensal bacterial consortium is obliterated with antibiotic treatment, pathogens can quickly take over the living space of intestinal tract, recruiting and aggregating with cooperative bacteria by QS, and ultimately construct a pathogen-dominated polymicrobial biofilm. In a notable study, the visualization of *E. coli* aggregates in murine intestinal contents has provided the first *in vivo* evidence of how chemotaxis toward AI-2 facilitates ecological niche segregation and stable co-existence of different *E. coli* strains.^91^ This study sheds light on the important role of QS in shaping bacterial communities in a living host. Taking *F. nucleatum* as another example, it can induce biofilm growth of single and dual species and coaggregation between itself and the “red complex”, which suggests the QS signal AI-2 plays an important role in inter- and intraspecies interactions between periodontopathogens.^92^ Whilst in the intestinal environment, *F. nucleaum* can aggregate with *C. difficile* and synergistically enhance the robust biofilm formation, however, whether QS involved in the process remains to be explored.^93^.

Once a biofilm dominated by pathogens is formed, the infectious healing will be negatively affected. Biofilms provide their inhabitants with competitive advantages, like efficient nutrients exchanging and increased resistance to environmental stress.^94^ Members in biofilm communicate and interact more frequently using QS due the proximal distance, thus drive collective behavior resulting in distinct physiologies from planktonic cells, like enhanced expression of virulent factors.^95^ Meanwhile, the horizontal gene transfer is promoted in biofilm microbiota, which contributes to spreading of antibiotic-resistance genes.^96^ In addition to enhancing information exchange and collaboration, the biofilm itself is a dense physical barrier rich in proteins and sugars that protects the bacteria inside from antibiotic, antimicrobial substances and host immune system, thereby exacerbating the infection.^97,98^ In addition, QS molecules secreted by bacteria are more likely to be retained in compact and hydrophobic biofilm, and then regulate host immune response by indirectly regulating the function of related microflora or directly acting on host cells.^95^ It has accepted that the pathogen-driven polymicrobial biofilm appears to be a tipping point between a healthy and diseased state of gut mucosa. Biofilms have been frequently recognized in several conditions, such as gut wounds,^99,100^ IBD,^101,102^ and CRC,^103,104^ which are supposed to participate in the pathogenesis and disease manifestation.^105,106^

QS signaling molecules, including AHL, AI-2, and AIP are reported possessing immunomodulatory effect on host tissues (Figure 2). As illustrated in the former section, 3OC12-HSL demonstrates immunomodulatory activities on T lymphocyte, macrophage and antibody responses in mammalian cells,^52,66^ while it can also suppress immune activity by inhibiting cytokine production and lymphocyte proliferation,^50,51^ disrupting NF-κB functions,^52^ and inducing immune cell death.^53^ AI-2 can affect the proinflammatory responses of host to GI pathogens, like *F. nucleatum*,^69^ *P. gingivalis*,^70^ and *H. pylori*.^56^ AI-2 could induce IL-8 expression in HCT-8 colon cancer cells.^54^ The autoinducing peptides (AIPs) synthesized by human microbiota were found to influence mammalian cells, promoting metastasis of cancer cells^57,60^ and penetrate the blood-brain barrier.^57^ Although the specific mechanism(s) through which AIs influence mammalian cells is unclear, modified immune responses by AIs unravel another circuit of how pathogens establish an infection.^107^

## Leveraging QS to manipulate the microbiota

### Quorum sensing interference as an anti-infectious therapy

Given the association between pathogens and the role they play in pathogenic process, QSI strategy, for instance, using QS signaling molecule analogues or degradation enzymes, has been raised as a promising therapeutic strategy replacing antibiotic to migrate microbial infections and resistance from human host. Up to date, plenty of efficient QSIs derived from food and plant that target human pathogens have been discovered using model pathogens by *in vitro* and *in vivo* animal models (Table 2). For example, some natural organic acids, fatty acids, vitamin C, furanone, flavone and its derivations perform anti-QS activities due to their structural similarity to QS signaling molecules.^130,131^ These QSI agents derived from plant-based foods, once taken into human body, are hypothesized to participate in the homeostasis maintenance of GI microbial communities by inhibiting pathogens’ QS signaling to avoid invasion. This may be one of the key reasons why the risk of intestinal inflammation is generally lower with plant-based foods compared to meat-based foods.^132^ However, to our knowledge, no direct evidence has been raised proving the validity of QSI strategies in protecting microbial barrier in human gut. *In vivo* test in murine infection models demonstrated the efficiency of QSI agent in protecting host skin barrier integrity by against *S. aureus*,^133^ which shows us the great potential for QSI to be used in the treatment of infectious diseases in mammalian host. We envision that the development of food-based anti-infection strategies targeting pathogens’ QS systems is a promising adjuvant or alternative to antibiotic.

**Table 2.** Efficient QSIs derived from food and plant that target human pathogens by in vitro and in vivo animal models.

QSI agent	Derivations	QS system affected	Function site	Bacteria affected	Function	Model	References
Oleic acid	Naturally present in staphylococcal abscesses and on the skin surface	/	/	*Staphylococcus aureus*	Inhibited primary adhesion	*In vitro*	^108^
Oleic and linoleic acids	Endophytic fungus *Arthrographis kalrae* isolated from *Coriandrum sativum*	/	/	*Streptococcus mutans*	Anti-biofilm	*In vitro*	^109^
Ascorbic acid (vitamin C)	/	AHL	LasI, lasR, rhlI, rhlR, pqsR and pqsA	*Pseudomonas aeruginosa*	Anti-virulence; anti-EPS	*In vitro*	^110,111^
Benzamine benzimidazole	Chemistry and Cell Biology (ICCB)-Longwood screening facility	AHL	Transcription factor MvfR (PqsR)	*P. aeruginosa*	Alleviate intestinal inflammation with redueced ileal TNFα and fecal lipocalin2 concentrations	*In vivo* (C57BL/6 mice)	
Coumarin	Cinnamomum verum	AHL	PqsA, rhlI, lasI	*P. aeruginosa*	Inhibit biofilm, phenazines production and swarming motility	*In vitro*	^112^
Furanones	Natural and synthetic derivatives	AHL	LasA, lasB and several other genes involved in virulence factor production	*P. aeruginosa*	Anti-virulence; anti-biofilm	*In vivo* and *in vitro*	^113^
Oleic acid	Rhizospheric bacterium *Stenotrophomonas maltophilia* BJ01	AHL	/	*P. aeruginosa*	Anti-biofilm	*In vitro*	^114^
Palmitoleic and myristoleic acids	/	AHL	Regulator *abaR*	*Acinetobacter baumannii*	Anti-biofilm	*In vitro*	^115^
Reb A/stevioside	Stevia extracts	AHL	Las/Rhl systems	*Escherichia coli*; *P. aeruginosa*	Inhibit bioluminescence emission	*In vitro*	^116^
Dihydrocoumarin	Coumarin	AHL	/	*Hafnia alvei*	Anti-biofilm	*In vitro*	^117^
Brominated furanone	Brominated, halogenated and natural forms in seaweeds, tomatoes, strawberries, and other berries; synthetic derivatives	AI-2	/	*Vibrio harveyi*, *Vibrio parahaemolyticus*	Reduce infection in gnotobiotic brine shrimp by pathogen *Vibrio*	*In vivo* (gnotobiotic brine shrimp *Artemia franciscana*)	^118^
Furanones	Synthetic derivatives	AI-2	/	*Listeria monocytogenes*	Anti-biofilm	*In vitro*	^119^
D-ribose	/	AI-2	/	*P. gingivalis* and *F. nucleatum*	reduction of alveolar bone loss	*In vivo* (BALB/c mice)	^120^
Malic acid	/	AI-2	/	*E. coli*; *Salmonella typhimurium*	Inhibit AI-2 activity	*In vitro*	^121^
Palmitic acid, stearic acid, oleic acid, linoleic acid	Ground beef extracts	AI-2	/	*V. harveyi*, *E. coli*	Inhibit AI-2 activity	*In vitro*	^122^
Ascorbic acid (vitamin C)	/	AI-2	Biofilm forming genes (fimA, csgA, luxS, malA and bssR)	*E. coli*	Anti-biofilm	*In situ*	^123^
Arachidonic Acid	Host-derived and dietary polyunsaturated fatty acids	AI-3	FadR	Enterohemorrhagic *E. coli* (EHEC)	Inhibite attachment to epithelial cells and the formation of attaching and effacing lesions by EHEC	*In vitro*	^124^
A unique 9 amino acid AIP	Commensal coagulase-negative staphylococci (CoNS)	AIP	Agr system	*S. aureus*	Reduce skin damage caused by s. aureus	*In vivo* (a murine infection model)	
Ascorbic acid (vitamin C)	/	AIP	ComA	*Bacillus subtilis*	Anti-biofilm	*In vitro*	^111^
Synthetic DSF analogues	Synthetic analogues	DSF	Histidine kinase PA1396	*P. aeruginosa*	Reducing biofilm formation and antibiotic tolerance	*In vitro* and *in vivo* (a murine infection model)	^125^
Linoleic acid	Plant unsaturated fatty acid	DSF	RpfF, rpfC, and rpfG	*P. aeruginosa*	Inhibit or induce the dispersion of P. aeruginosa biofilms	*In vitro*	^126^
*Lactococcus lactis*	/	CAI-1	CqsS	*Vibrio cholerae*	Cholera diagnosis	*In vivo*	^127^
*E.coli*	/	CAI-1	CqsS	*V. cholerae*	Cholera diagnosis	*In vitro*	^128^
Mammalian cell	/	AI-2	FPR1protei	*V. harveyi* and *Candida albicans*	Anti-biofilm	*In vitro*	^129^

### Engineering the QS systems of probiotics and symbiotic bacteria for disease diagnosis and treatment

**Figure 4.**
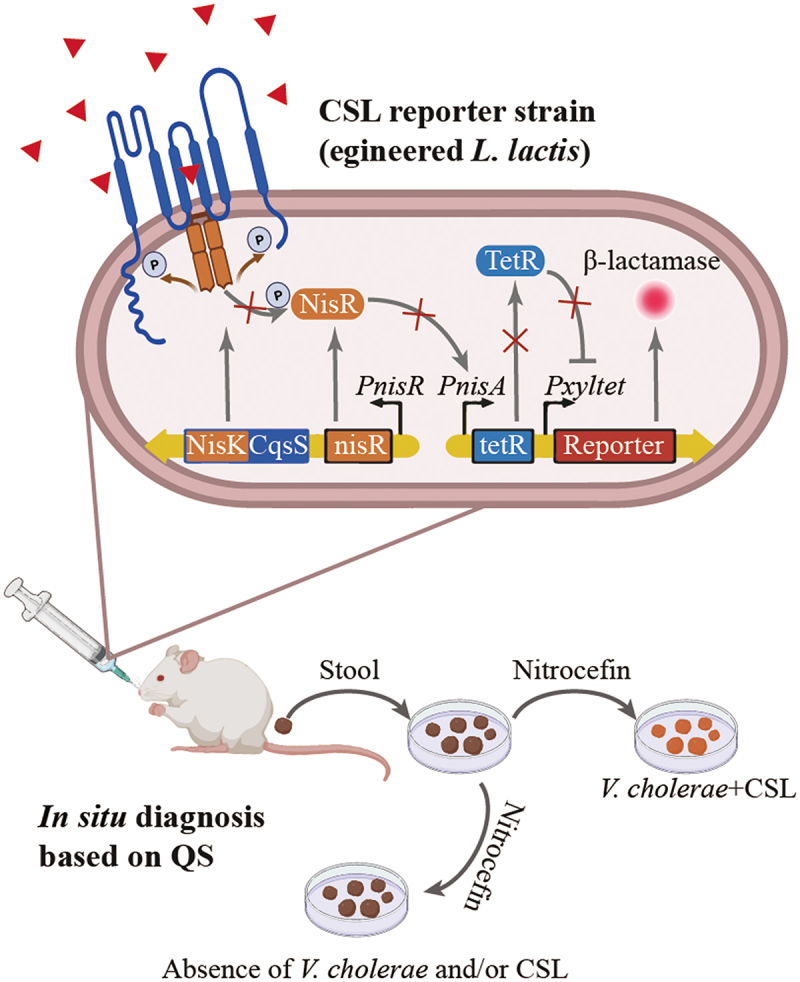
Leveraging QS to manipulate the microbiota.

In addition to the usage of QSI analogs to mitigate virulence from infectious pathogens, more studies try to design and engineer the QS pathway of probiotics and symbiotic bacteria for disease diagnosis and treatment. Mao et al.^127^ and Holowko et al.^128^ made the first attempt using engineered probiotic *Lactococcus lactis* and nonpathogenic *E.coli* to mediate cholera resistance in animal models. Holowko created a synthetic genetic CAI-1 sensing system with a sensor module and an inverter module. In the absence of quorum-sensing molecule CAI-1 that unique to the genus *Vibrio*, gene cassette, including CqsS, LuxU and LuxO are constitutively expressed and phosphorylated, thereafter, the phosphorylated LuxO activates Qrr4 promoter which expresses gRNA to further joins constitutively expressed dCas9 to repress GFP expression. While when *V. cholerae* reaches a high-density state, CAI-1 dephosphorylates CqsS will ultimately dephosphorylates LuxO. Dephosphorylated LuxO does not activate the Qrr4 promoter preventing gRNA expression therefore GFP can be expressed efficiently without gRNA inhibition. In the latter study, Mao advanced the QS based diagnosis *in vitro* close to the stage for dietary interventions with natural and engineered probiotics as an alternative strategy to combat the spread of cholera in vulnerable populations using a probiotic *L. lactis* (Figure 4). The engineered *L. lactis* can detect *V. cholerae* and trigger expression of its enzymatic reporter that is readily detected *in situ* fecal samples. Such living diagnostics based on bioengineering of QS represents a promising approach to achieve near real-time surveillance of multiple pathological conditions.^127^ Recently, there have been significant advances in the engineering of QS devices for dynamically controlling bacterial populations and developing potential clinical therapies. Wu and Dang have conducted comprehensive reviews of these recent achievements.^134,135^

Cholera sensing and reporting by engineered *L. lactis* (CSL) in situ and in vivo. In the present of CAI-1, the hybrid two-component system (Nisk-CqsS encoded sensing module) stops its phosphorelay, halting TetR expression and leading to activation of the production of β-lactamase (reporter gene). On sensing CAI-1, the *L. lactis* CSL strain secretes lactamase, which hydrolyzes nitrocefin and produces a visible color change from yellow to red.

Complementary to the living therapies based on bacterial interactions, Sedlmayer and his colleagues designed a synthetic mammalian cell (human HEK-293 cell) based microbial-control device that detects microbial chemotactic formyl peptides through a formyl peptide sensor (FPS) and responds by releasing AI-2.^129^ In details, N-formyl peptides activate the sensor FPR1, which was rewired to the constitutively expressed promiscuous human G protein subunit Gα16 and relays to calcium-triggered expression of LuxS. In the assistance with MTAN, LuxS generates AI-2 which can be further released outside the mammalian cell and sensed by AI-2 responsive pathogens, such as *V. harveyi* and *Candida albicans*. The biosensor-based circuits in implanted human designer cells programmed to interface with infection-related molecules and to mitigate microbial colonization may pave the way for next-generation antimicrobials in the post-antibiotic era.^129,136^

### Preliminary attempt to shape the assembly of human microbiota by manipulating QS communication network

In recent years, more research tries to exploit novel strategies to promote gastrointestinal homeostasis from the aspect of microbiota-modulating. Because of the large diversity of bacteria in the gut, multiple QS pathways have been mined out by *in silico* search.^137^ These QS systems are suspected to play vital roles regulating microbe-microbe interactions to maintain the harmonious of human gut microbiome.^6,138^ In the previous study, Thompson et al. used an engineered *E. coli* to restore the level of AI-2 in antibiotic-treated mice, and ultimately relanced the ratio of *Bacteroidetes* to *Firmicutes* phyla in the murine gut bacterial community,^38^ which demonstrated the AI-2 availability in shaping the antibiotic-ruined GI microbiota. Nevertheless, very little is currently known with respect to the role of QS in complex microbial communities and their use in the detection, prevention, and treatment of acute infections remains underexplored. In our recent work (unpublished), we developed and *in vitro* multispecies (more than 300 species) oral biofilm assembly model, based on which attempting to revealing the shaping power of QS network in the assembly process by performing time serial metagenomic analysis and QS-interfering experiment. We constituted a full-scale longitudinal QS network via the QS hubs of *Streptococcus*, the *Veillonella-Megasphaera* group, and the *Prevotella-Fusobacteria* group for information delivery, and experimentally validated the directional shaping power of the longitudinal QS network in the microbiota. This work attempts to illustrate the role of longitudinal QS network in shaping human microbiota assembly, which could lead to more in targeted interventions for manipulating human microbiota through QS network intervention.

### Challenges in applying QS based interventions for microbiota manipulation

Before the widespread adoption of QS therapies in the treatment of individuals, there are still several overarching challenges that need to be addressed. The first significant obstacle is assessing how QS functions in host environments that involve fluid flow and complex geometries. Flow is present in all living systems, such as, intestinal digestion and urination. Flow environment drives heterogeneously distributed QS signals in specific locations, therefore leading to spatial fate decisions which allows genetically identical bacterial to undertake distinct biological functions.^8,15,95^ Additionally, surface topography is another realistic factor influencing QS dynamics, which is constantly found to drive non-uniform bacterial behavior conjunction with flow. Kim et al. applied microfluidic chambers with cervices to mimic intestinal crypts, revealing the heterogeneous QS activities in various localized bacteria.^95^ Outside of the crevices, the QS activity were repressed due to constant flow, while bacteria that colonized inside the crevices exhibited heightened QS activity in response to signal accumulation. It is crucial to investigate how QS is deployed under realistic circumstances, such as clinical and industrial settings.

QS as a mechanism that regulate communication and gene expression in bacteria, plays a crucial role in the mutual antagonism between probiotics and pathogens in the gut microbiota. When probiotics detect the presence of pathogens in their environment, they can release signaling molecules to inhibit the QS system of the pathogens, suppressing their growth and the production of virulence factors. This competition slows down or prevents the pathogenicity of the pathogens and contribute to maintain a healthy balance in the gut. However, some pathogens can also utilize the QS system to counteract probiotics. In response to the presence of competitors like probiotics, pathogens can adjust gene expression, produce antimicrobial substances, or generate toxic metabolites to inhibit the growth and activity of probiotics. This antagonistic behavior helps pathogens establish a competitive advantage in the gut and enhances their ability to infect the host. Under normal circumstances, there is a dynamic equilibrium between probiotics and pathogens. This balance helps to maintain a stable gut environment, preventing the excessive proliferation of pathogens and protecting the host from disease invasion. However, deciphering how probiotics and pathogens decode the information in different combinations of signaling molecules produced by cooperating and competing bacteria remains a significant challenge.

While bacterial infections are well-known, it is important to note that fungal infections of the gastrointestinal tract can also occur, especially in individuals with compromised immune systems or other underlying health conditions. One of the most common fungal species involved in gastrointestinal infections is *Candida*, specifically *Candida albicans*. *C. albicans* utilizes QS based on farnesol to mediate metabolisms, such as morphogenesis, biofilm development, mating, drug efflux, and apoptosis.^139–143^ QS in *C. albicans* also plays a role in inter-kingdom crosstalk, such as repressing the production of the quinolone QS signal in *P. aeruginosa* ^144^ and triggering the release of neutrophil extracellular traps.^145^ These findings indicates that QS communication is involved in a broad range of interactions within microbiota and between microbes and the host. It is crucial for future studies to explore QS mechanisms regarding more realistic contexts, including the presence of flow and the inclusion of fungi, viruses and eukaryotic hosts, to further enhance our understanding and application of the QS communication mechanism.

## Concluding remarks and future considerations

The misuse and overuse of antibiotics have led to the emergence of multidrug-resistant bacterial strains, posing a global health threat and limiting the effectiveness of conventional antibiotic treatments. As a result, researchers have been actively exploring new strategies to combat bacterial infections. Quorum sensing (QS) has emerged as an attractive target due to its regulatory effects on bacterial virulence. In recent decades, numerous studies have summarized and demonstrated the QS-based approaches alleviating and preventing infections.^134,135,146,147^ The growing number of studies that focus on QS-based applications shows a bright future for this field. However, our understanding of how to leverage QS to precisely manipulate microbiota is still in the early stage, and there are still many significant challenges that need to be addressed. First, the complex and dynamically changing environment can significantly impact the QS signaling cascade, leading to a heterogeneity in microbiota using QS regulatory pathway. Therefore, it is crucial to investigate how QS is deployed in realistic scenarios, such as clinical and industrial settings, which involve fluid flow and complex geometries. Second, understanding how probiotics and pathogens decode the information contained in blends of AIs produced by cooperating bacteria versus competing bacteria is essential. This knowledge is vital for selecting appropriate QS based interference strategy to target specific pathogens without interfering with the QS network inherent in the symbiotic microbiota. This is crucial in order to avoid causing undesirable flora dysbiosis and diseases. Third, the QS regulatory mechanisms not only facilitate communication among bacterial populations, but also play a role in the cross-talk between bacteria, fungi, and the mammalian host. Therefore, further investigations are urgently needed to decipher the QS molecules and pathways involved in inter-kingdom communication. Finally, to ensure the efficiency and safety of QS based applications, it is necessary to conduct more *in vivo* test rather than relying solely on *in vitro* experiments. To sum up, future studies exploring the QS communication network in complex, natural, multispecies consortia will enhance our understanding of the mutualistic symbiotic relationship between the microbiota and the host. This knowledge will provide opportunities for the development of more precise therapeutics to combat bacterial infections and enhance host immune defenses.

## Data Availability

Data sharing is not applicable to this article as no new data were created or analyzed in the review.
